# Independent role of caspases and Bik in augmenting influenza A virus replication in airway epithelial cells and mice

**DOI:** 10.1186/s12985-023-02027-w

**Published:** 2023-04-24

**Authors:** Sourabh Soni, Stephanie Walton-Filipczak, Richard S. Nho, Yohannes Tesfaigzi, Yohannes A. Mebratu

**Affiliations:** 1 Division of Pulmonary, Critical Care, and Sleep Medicine, Department of Internal Medicine, The Ohio State University Wexner Medical Center, Columbus, OH USA; 2 Lovelace Respiratory Research Institute, Albuquerque, NM USA; 3 New Mexico Department of Game and Fish, Santa Fe, NM USA; 4 Division of Pulmonary and Critical Care Medicine, Department of Medicine, Brigham and Women’s Hospital, Harvard Medical School, Boston, MA USA

**Keywords:** Influenza A virus, Caspase, Q-VD-Oph, PARP1, *Bik* wild-type and knockout mice

## Abstract

Caspases and poly (ADP-ribose) polymerase 1 (PARP1) have been shown to promote influenza A virus (IAV) replication. However, the relative importance and molecular mechanisms of specific caspases and their downstream substrate PARP1 in regulating viral replication in airway epithelial cells (AECs) remains incompletely elucidated. Here, we targeted caspase 2, 3, 6, and PARP1 using specific inhibitors to compare their role in promoting IAV replication. Inhibition of each of these proteins caused significant decline in viral titer, although PARP1 inhibitor led to the most robust reduction of viral replication. We previously showed that the pro-apoptotic protein Bcl-2 interacting killer (Bik*)* promotes IAV replication in the AECs by activating caspase 3. In this study, we found that as compared with AECs from wild-type mice, *bik*-deficiency alone resulted in ~ 3 logs reduction in virus titer in the absence of treatment with the pan-caspase inhibitor (Q-VD-Oph). Inhibiting overall caspase activity using Q-VD-Oph caused additional decline in viral titer by ~ 1 log in *bik*^*-/-*^ AECs. Similarly, mice treated with Q-VD-Oph were protected from IAV-induced lung inflammation and lethality. Inhibiting caspase activity diminished nucleo-cytoplasmic transport of viral nucleoprotein (NP) and cleavage of viral hemagglutinin and NP in human AECs. These findings suggest that caspases and PARP1 play major roles to independently promote IAV replication and that additional mechanism(s) independent of caspases and PARP1 may be involved in Bik-mediated IAV replication. Further, peptides or inhibitors that target and block multiple caspases or PARP1 may be effective treatment targets for influenza infection.

**Supplementary Information:**

The online version contains supplementary material available at 10.1186/s12985-023-02027-w.

## Background

Influenza A virus (IAV) infection has resulted in 9 to 41 million illnesses, 140,000 to 710,000 hospitalizations, and 12,000 to 52,000 deaths annually between 2010 and 2020 in the United States, which translates into economic losses worth tens of billions of dollars [1]. The most recent and the first flu pandemic of the 21st century caused by the H1N1/pdm09 virus was reported in 2009 in 207 countries and resulted in 42 to 86 million infections [2]. This IAV has continued to spread as a seasonal epidemic which significantly impacts global health with an annual burden of around 3 to 5 million cases of severe illness and about 290,000 to 650,000 mortalities worldwide [3]. Additionally, a highly pathogenic avian H5N1 IAV has spread throughout Africa, Asia, and Europe by overcoming host species barriers to infect humans and cause mortalities [4, 5]. IAV evolves to escape humoral immunity *via* mutations in hemagglutinin (HA) and neuraminidase (NA) epitopes to circumvent the antibodies induced through past infections and vaccinations [6]. Frequent mutations in IAV genes necessitates the generation of new vaccines annually, which is an expensive endeavor and does not necessarily offer sufficient protection. Further, the rapid evolution of viral genomes towards resistance poses serious challenges for effective therapeutic interventions. Therefore, identifying host cellular proteins required for IAV replication, and to exert its pathogenicity, is crucial for designing effective antiviral drugs that are less susceptible to resistance.

IAV induces apoptosis in infected epithelial, lymphocyte, and phagocytic cells [7] and mainly damages epithelial cells of the human respiratory tract [8]. After infection, IAV proteins interact with host proteins and use the host cellular machinery for virus replication [9, 10]. Host cellular proteases including the ones activated by pro-apoptotic members of the Bcl-2 family proteins, mainly the **C**ysteine **A**spartate-**S**pecific **P**rote**ases** (caspases) and the substrate of caspases, poly (ADP-ribose) polymerase (PARP1), promote IAV replication through proper assembly and maturation of viral proteins [11–16].

Caspases are members of the cysteine proteases and play crucial roles in controlling host cell death, innate immune responses, and homeostasis [17, 18]. IAVs express several proteins that are cleaved by host cell caspases [19]. Almost all IAV proteins contain caspase cleavage motifs, and these motifs are recognized and specifically cleaved at the D amino acid (aspartic acid) by the host cell proteases [20]. Notably, several caspases target identical cleavage motifs on the IAV proteins, and many viral proteins share similar caspase cleavage motifs [13]. For example, IAV nucleoprotein (NP) and matrix protein 2 (M2) possess caspase cleavage motifs METD_16_↓G at the N-terminus and VDVDD_87_↓G at the C-terminus, respectively, which are cleaved at the late apoptotic stage in the infected cells [13]. These caspase motifs may be involved in virus replication and in host pathways of infection immunopathogenesis [19]. The role of caspases, mainly caspase 3 [11, 12, 21, 22] and their downstream substrate PARP1 [14] in IAV replication and pathogenicity has been studied in various disease models. However, there is very limited data available on the role of caspase 2 and 6 on IAV replication in the airway epithelial cells (AECs), and to date, direct comparison of the effect of the individual caspases in IAV replication in the AECs has not been reported. Further, the exact role of PARP1 in regulating viral replication remains obscure.

In addition to their role in processing IAV proteins and the maturation of viral particles [23], IAV-induced caspases are also responsible for widening of nuclear pores to facilitate passive transport of viral ribonucleoprotein (vRNP) to ensure efficient production of infectious virus progeny [24, 25]. IAV NP acts as a shuttle for viral genomic segments from the nucleus to the budding sites at the plasma membrane and reduced cytoplasmic translocation of NP during the virus replication cycle affects virus titers. Following apoptotic stimuli, vRNP or NP translocate partially to the cytoplasm in a caspase 3-dependent manner [11], and lack of caspase activity leads to nuclear retention of NP and decreased viral replication [26]. Pro-apoptotic members of the Bcl-2 family proteins activate PARP1 through caspase 3 and 7 in cell culture and in vivo [27, 28]. During apoptosis, PARP1 is cleaved into two fragments, the amino-terminal DNA-binding domain (24 kDa) and the carboxy-terminal catalytic domain (89 kDa) [27, 29]. PARP1 regulates IAV polymerase activity [30, 31] and controls viral HA-induced degradation of type I interferon receptor (IFNAR), a subversion tactic of IAV to evade host immunity and accelerate viral replication [16].

We previously showed that viral replication was diminished in *bik* knockout (*bik*^*−/−*^) mouse airway epithelial cells (MAECs). Furthermore, *bik*^*−/−*^ mice were resistant to a lethal dose of IAV infection. IAV-induced Bik activated caspase 3 and led to the cleavage of viral NP and M2 proteins [12]. However, whether other cysteine proteases not activated by Bik or whether additional mechanism(s), likely mediated by PARP1 are involved in Bik-mediated replication of IAV is not clear. Protease inhibitors have recently been demonstrated to be effective tools for the treatment of viral infections, such as hepatitis C virus (HCV) and human immunodeficiency virus-1 (HIV-1). HIV-1 protease inhibitors are among the most effective antiretroviral drugs used successfully to prevent cleavage of HIV-1 viral proteins, resulting in superior antiviral activity [32]. The HCV specific protease inhibitors that block the enzymatic activity of the HCV nonstructural 3 (NS3) protease region necessary for protein processing and viral replication leads to marked decrease in HCV replication and eradication of the infection in patients with chronic hepatitis C [33, 34]. However, to date, no specific host cell protease inhibitors have been identified that are effective therapeutic tools to treat IAV infection. Here, we evaluated individual cysteine proteases and PARP1 for their role in IAV replication in AECs and show that, in addition to caspase 3; caspases 2 and 6, and PARP1 play major roles to independently promote IAV replication. Furthermore, we showed that overall inhibition of caspase activity protects mice from IAV-induced lung inflammation and lethality.

## Methods

### Animals

Six to eight weeks old wild-type (WT) C57BL/6 mice were purchased from Jackson Laboratory (Bar Harbor, ME, USA). The *bik*^*-/-*^ mice on C57BL/6 background were provided by Andreas Strasser (Walter and Eliza Hall Institute, Melbourne, Australia) and housed and bred following set guidelines at our institutional animal facility and genotyped as described previously [35] and used to isolate MAECs. Both male and female mice were used in this study. All experiments were approved by the Institutional Animal Care and Use Committee and the Institutional Environmental Safety and Health Department of Lovelace Respiratory Research Institute.

### Cell culture

Primary MAECs isolated from mouse trachea and immortalized human airway epithelial cells (HAECs) provided by S. Randell (University of North Carolina, Chapel Hill, NC, USA) described previously [36] were cultured on Transwell membranes (Corning, New York, NY, USA) as described previously [37]. The primary cells were cultured in bronchial epithelial growth medium (BEGM) and small AEC growth media (SAGM) (Lonza, Basel, Switzerland), respectively, supplemented with growth factors (BEGM and SAGM Singlequots; Lonza) and incubated at 37 °C under an atmosphere containing 5% carbon dioxide as described [38]. MAECs were isolated from murine tracheas after overnight digestion with pronase, washing in phosphate buffered saline (PBS) and were cultured as previously described [39].

### Air-liquid interface (ALI) culture

Cells were seeded on petriplates and grown to 60–70% confluency and ∼5 × 10^5^ cells were transferred onto 12-well Transwell membrane (Corning) to allow differentiation in an air-liquid interface condition. Once cells reached complete confluence, the medium from the top compartment was removed and cells were fed only from the bottom compartment to allow the ALI differentiation to occur over a 3 weeks period. The medium was changed every other day.

### Measurement of trans-epithelial electrical resistance (TEER)

TEER developed by the differentiated airway epithelial cell cultures was measured by using the Millicell® ERS-2 Voltohmmeter (Millipore, MA, USA) according to the manufacturer’s instructions.

### Viral growth and titer determination

IAV A/Puerto Rico/8/1934 (H1N1) strain (PR/8) was propagated in embryonated chicken eggs and titrated on Madin Darby canine kidney (MDCK) cells grown in minimum essential medium (MEM) (Invitrogen, Waltham, MA, USA) as described [7]. Primary MAECs were allowed to differentiate in ALI and infected from apical side with mock or 0.1 MOI of the virus for 1 h followed by washing with PBS. Further, fresh medium was added, and cells were incubated at 37 °C. Infectious virus yields were analyzed from the apical washes (200 µl) collected 48- or 72-hours post-infection (hpi) *via* plaque assay as described [12]. TPCK-Trypsin was added in the viral infection media (working concentration 3 µg/ml). Briefly, monolayers of MDCK cells were cultured overnight in Dulbecco’s Modified Eagle Medium (DMEM) containing 10% fetal bovine serum (FBS) and 1% penicillin-streptomycin and infected with 10-fold serial dilutions of virus suspension for 1 h at room temperature (RT). Cells were then covered with warmed Eagle’s MEM containing 0.1% DEAE dextran and 1% purified agar. The agar medium was allowed to solidify at RT and incubated for 2 to 3 days at 37 °C to promote plaque development. Immediately, prior to plaque analysis, the solidified agar was removed, and cells were fixed and stained with a methanol-crystal violet solution. Plaques were counted, and the virus titer was expressed as plaque-forming unit (pfu)/ml.

### Viral titers in lung tissues and the median tissue culture infectious dose (TCID_50_) assay

The mouse lungs were harvested at 5 days post-infection (dpi) and homogenized using a tissue homogenizer (Omni International, Kennesaw, GA, USA) as previously described [40]. The homogenates were centrifuged at 1500 g for 10 min at 4 °C. The supernatants were collected and separated into aliquots. Virus titer was determined immediately. For TCID_50_ assay, confluent monolayers of MDCK cells in 96-well plates were inoculated with 10-fold dilutions of the samples (8 wells per dilution) and incubated for 3 days; wells showing positive cytopathic effects were counted, and the TCID_50_ titer was interpolated using the Reed-Muench method [41] as described before [42]. The lowest detection limit of the TCID_50_ assay was approximately 10^2.

### Lung histopathology

The lung tissues were fixed in 10% formalin, embedded in paraffin, cut, and stained with hematoxylin and eosin to analyze inflammation-associated lung damage. Histopathologic inflammation score of lung tissues was evaluated in a blinded manner and scored with a semi-quantitative system according to the relative degree of inflammation and tissue damage. Lung inflammatory changes were graded based on the following parameters: peribronchiolar and bronchial infiltrates, bronchiolar and bronchial luminal exudates, perivascular infiltrates, parenchymal pneumonia, and edema, as previously described [40, 43]. Each parameter was graded on a scale of 0–4 with 0, absent; 1, slight; 2, mild; 3, moderate; and 4, severe. The cumulative scores of inflammatory infiltration, degeneration, and necrosis provided the total score per animal, and the average score of mice in each group was taken as total score for that group.

### Caspase and PARP1 inhibition

In cells infected with IAV, caspase activities were inhibited using specific caspase inhibitors or a broad-spectrum caspase inhibitor. The following inhibitors were used in HAECs or *bik* WT (*bik*^*+/+*^) and *bik*^-/-^ MAECs infected with IAV: caspase 2 inhibitor Z-VDVAD-FMK: Z-V-D(OMe)-V-A-D(OMe)-FMK (#FMK003, R&D, Minneapolis, MN, USA); caspase 3 inhibitor Z-DEVD-fmk: Z-D(OMe)-E(OMe)-V-D(OMe)-FMK (#FMK004, R&D); caspase 6 inhibitor Z-VEID-FMK: Z-V-E(OMe)-I-D(OMe)-FMK (#FMK006, R&D); PARP1 inhibitor 4-AN: 4-Amino-1,8-naphthalimide (#4667-50-09, R&D); and broad-spectrum caspase inhibitor Q-VD-Oph: quinoline-Val-Asp-difluorophenoxymethylketone (#OPH001-01 M, R&D). Briefly, cells were seeded in 10 cm petri dishes, infected with 0.1 MOI IAV, and treated with vehicle control (PBS) or 20 µM of the caspase inhibitors. Our previous studies showed that 20 µM Q-VD-Oph is sufficient to inhibit caspase activations without perceivable toxicity to the cells [12]. Hence, we used 20 µM Q-VD-Oph in the in vitro experimentation in this study. To be consistent throughout the experimental groups, we used identical doses of all the inhibitors so that accurate comparisons could be drawn among them. Also, previous reports have shown the use of these inhibitors in culture at 20 µM dose [44–46]. The inhibitors were added on the apical and basal sides for  3 h prior to viral infection following which the apical media was removed and the inhibitor containing media was kept on the basal side until harvest for analysis. 72 hpi apical washes and cell extracts were harvested to analyze viral titers. For in vivo application, the recommended dose is 20 mg/kg, and doses up to 120 mg/kg have been reported to cause no toxicity in mice [47]. Therefore, at day − 1 and day 1 prior to intranasal instillation of 100 pfu PR/8 in 50 µl PBS, mice were injected intraperitoneally (IP) with vehicle (PBS) as control or 20 mg/kg Q-VD-Oph. Isoflurane was used as an anesthetic for virus instillation of the mice. Mice were monitored daily for a period of 14 days for signs of disease, changes in body weight, and survival. Mice were euthanized when moribund and at the end of the study (14 dpi) using pentobarbital. The humane endpoint governing euthanasia of animals during viral challenge studies was the loss of 20% of the original body weight of mouse. Lungs were formalin inflated and used for histopathological analysis to assess lung inflammation. For histopathology and viral titers, we used lung specimens from different animals of the same experimental group.

### Immunoblotting

Protein lysates were prepared and analyzed by Western blot as described previously [48]. Briefly, to analyze protein expression following appropriate treatments the cultured cells were lysed using RIPA cell lysis buffer (Alfa Aesar, Haverhill, MA, USA) containing protease inhibitor cocktail (Sigma-Aldrich, St. Louis, MO, USA). Lysates were then subjected to SDS-PAGE and Western blot analysis for cleavage of caspases, PARP1, viral HA, and NP. The following antibodies were used: mouse anti-caspase 2, 1:1000 dilution (#2224, Cell signaling Technology, Inc., Danvers, MA, USA), rabbit anti-caspase 3, 1:1000 dilution (#9661, Cell Signaling Technology, Inc.), rabbit anti-caspase 6, 1:1000 dilution (#9761, Cell Signaling Technology, Inc.), anti-PARP1, 1:1000 diution (#5625, Cell Signaling Technology, Inc.), mouse anti-NP antibody, 1:1000 dilution (#GTX00858, GeneTex, Irvine, CA, USA), and rabbit anti-HA antibody, 1:500 (#SAB3500061, Sigma-Aldrich). Equal protein loading was confirmed by probing with the mouse monoclonal antibody against actin (1:10,000 dilution) from Santa Cruz Biotechnology, Inc. (Santa Cruz, CA, USA). Peroxidase AffiniPure goat anti-mouse IgG (#115-035-003) and anti-rabbit IgG (#111-035-003), Jackson ImmunoResearch Laboratories, Inc., West Grove, PA, USA were used as secondary antibodies at 1:10,000 dilution. Specific protein expression was detected and visualized using enhanced chemiluminescence (ECL) substrate (Thermo Scientific, MA, USA) with ChemiDoc imaging system (Biorad, CA, USA). For quantification of protein bands ImageJ software version 1.53t (NIH, MD, USA) was used.

### Immunostaining

HAECs were grown on chamber slides (Corning). Following infection with IAV and treatments with Q-VD-Oph, cells were fixed with 4% paraformaldehyde containing 3% sucrose in PBS for 15 min, permeabilized with 0.25% Triton X-100 for 10 min and blocked with 3% BSA for 1 h at RT. The cells were then incubated overnight at 4°C with anti-NP antibody followed by secondary antibody conjugated to Dylight-649 for 1 h at RT. The cells were then mounted with DAPI-containing Fluormount-G (Southern Biotech, Birmingham, AL, USA) for nuclear staining. Immunofluorescence was imaged using Axioplan 2 microscope (Carl Zeiss, Inc., Thornwood, NY, USA) with a PlanNeofluor 403/0.75 air objective and a charge-coupled device camera (Hamamatsu Photonics, Hamamatsu, Japan) with the acquisition software Slidebook 5.0 (Intelligent Imaging Innovation, Denver, CO, USA).

### Data analyses

Data are expressed as the mean group value ± standard error mean (SEM) and analyzed using GraphPad Prism 9.4.1. (GraphPad Software Inc., CA, USA). Data from each group of treatment were subjected to two-tailed unpaired student’s *t* test. The criterion for significance was P < 0.05 in all studies. For the in vivo studies, differences in body weight were expressed as mean group value ± SEM. The survival rates of mice were analyzed using the log-rank (Mantel-Cox) test.

## Results

### Specific caspase and PARP1 inhibitors mitigate IAV replication in the airway epithelial cells

**Fig. 1:**
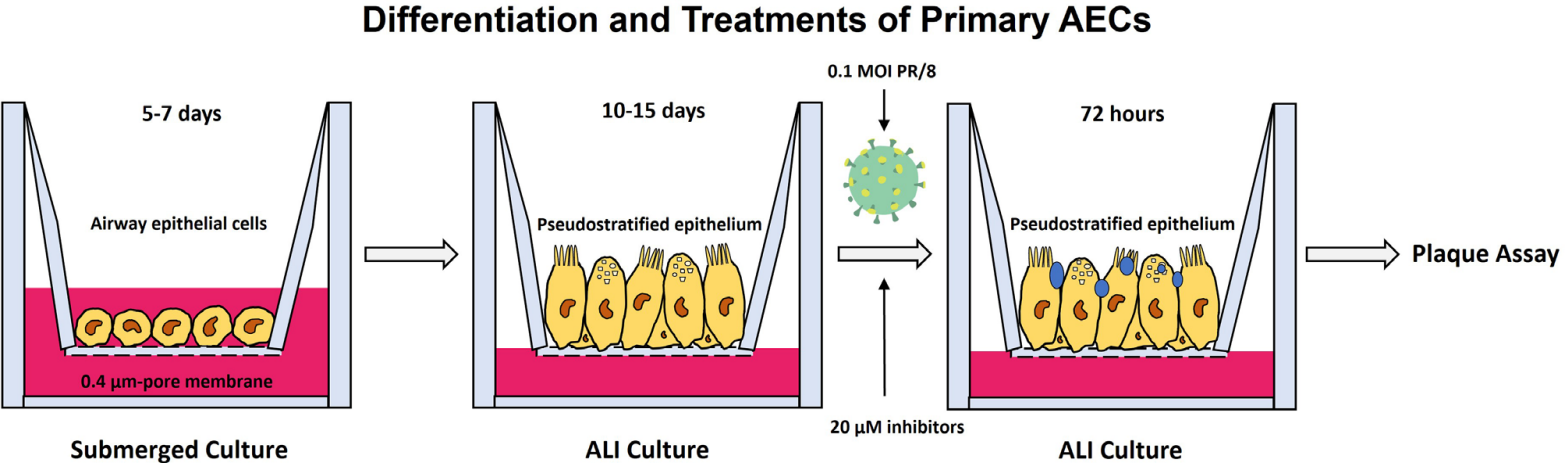
Schematic representation of infection and treatments of differentiated airway epithelial cells (AECs). Primary AECs were driven to differentiation in air-liquid interface cultures and infected with 0.1 MOI IAV (H1N1) PR/8/34 strain and treated with vehicle control or 20 µM caspase or PARP1 inhibitors on apical and basal sides 3 h prior to viral infection. The inhibitors were removed from the apical side but were kept with media in the basal side for the whole experimental period. Infectious virus yields were analyzed from the apical washes collected 48- or 72-hours post-infection (hpi) *via* plaque assay. The blue circles resting atop the cells in the right-most diagram depict mucus secreted by virus-infected differentiated AECs

**Fig. 2:**
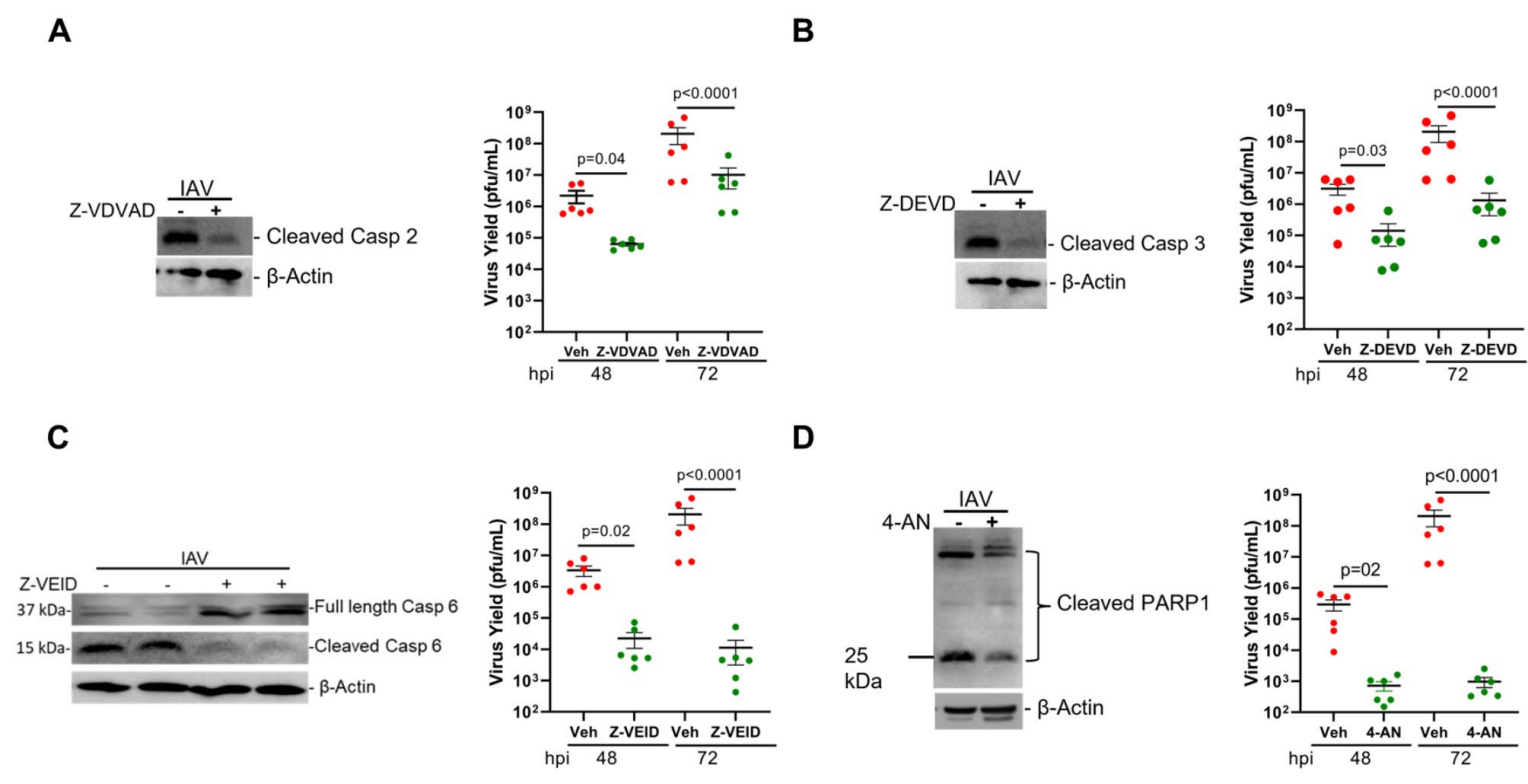
Specific caspase and PARP1 inhibitors mitigate IAV replication in the airway epithelial cells (AECs). Differentiated AECs were infected with 0.1 MOI IAV (H1N1) PR/8/34 strain and treated with vehicle only or 20 µM of **(A)** caspase 2 inhibitor, Z-VDVAD-FMK (Z-V-D(OMe)-V-A-D(OMe)-FMK); **(B)** caspase 3 inhibitor, Z-DEVD-fmk (Z-D(OMe)-E(OMe)-V-D(OMe)-FMK); **(C)** caspase 6 inhibitor, Z-VEID-FMK (Z-V-E(OMe)-I-D(OMe)-FMK); or **(D)** PARP1 inhibitor, 4-AN (4-amino-1,8-naphthalimide). The inhibitors were added on apical and basal sides 3 h prior to viral infection. The inhibitors were removed from the apical side but were kept with media in the basal side for the whole experimental period. Protein lysates were analyzed for caspase cleavages by Western blot. The infectious viral titers were analyzed from apical washes of the cells 48- and 72-hours post-infection using the plaque assay. Experiments were performed in triplicates and the immunoblots are representative of at least three separate experiments. Error bars indicate ± SEM, (n = 6/group for all experiments; N = 2); p < 0.05 was considered significant

Previous studies from our laboratory and others have shown that host cellular proteins, mainly caspase 3 and PARP1, promote IAV replication through proper assembly and maturation of viral proteins [11–16, 49]. To examine the role of caspases 2, 3, 6, and their substrate, PARP1, in promoting viral replication, we used specific inhibitors. Differentiated primary HAECs were infected with 0.1 MOI PR/8 and treated with vehicle control or 20 µM of caspase 2, 3, 6, or PARP 1 inhibitors. Forty-eight and seventy-two hpi the cell lysates and apical washes were analyzed for cleavage of proteins and viral titer, respectively **(**Fig. 1**).** We found that, compared with the vehicle-treated group, inhibition of caspase 2 caused approximately 1.5 log reduction in viral titer both at 48 and 72 hpi **(**Fig. 2A**)**, whereas treatment with caspase 3 inhibitor caused about 1.5 log reduction in viral titer 48 hpi and 2.5 log reduction 72 hpi **(**Fig. 2B**)**. Treatments with caspase 6 and PARP1 inhibitors led to the most significant decline in viral replication with caspase 6 inhibitor resulting in about 2 log reduction 48 hpi and 4.5 log reduction 72 hpi **(**Fig. 2C, Supplementary Figure S3) and PARP1 inhibitor causing about 3 log reduction 48 hpi and 6 log reduction 72 hpi, although PARP1 cleavage appeared to only be partially blocked **(**Fig. 2D**)**.

### Pan-caspase inhibitor diminished IAV replication in mouse airway epithelial cells in a Bik-independent manner

**Fig. 3:**
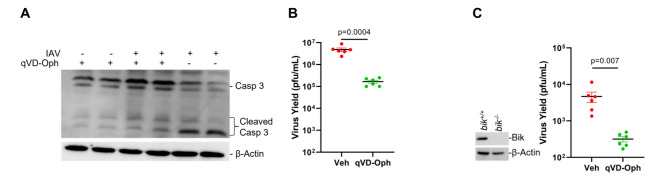
Pan-caspase inhibitor diminished IAV replication in mouse airway epithelial cells (MAECs). **(A)** Differentiated MAECs were infected with 0.1 MOI IAV (H1N1) PR/8/34 strain and treated with vehicle control or 20 µM of the pan-caspase inhibitor, Q-VD-Oph. The inhibitors were added on apical and basal sides 3 h prior to viral infection. The inhibitors were removed from the apical side but were kept with media in the basal side for the whole experimental period. Protein lysates were analyzed for caspase 3 cleavage by Western blot. The infectious viral titers were analyzed from apical washes of the cells 72 h post-infection in **(B) ***bik*^+/+^ cells, **(C) ***bik*^−/−^ cells. Protein lysates were analyzed for the expression of Bik by Western blotting. Experiments were performed in triplicates and the immunoblots are representative of at least three separate experiments. Error bars indicate mean ± SEM (n = 6/group for all experiments; N = 2); p < 0.05 was considered significant

Several caspases target identical cleavage motifs on the IAV proteins and many viral proteins share the same caspase cleavage motifs [13], suggesting that inhibiting multiple caspases may be required to attain effective inhibition of viral replication. Therefore, we used Q-VD-Oph, a broad-spectrum caspase inhibitor [49–52], to investigate whether loss of the overall caspase activity leads to efficient reduction in viral replication in the AECs. Our previous studies showed that 20 µM Q-VD-Oph is sufficient to inhibit caspase activations without perceivable toxicity to the cells [12]. Analysis of the protein lysates from differentiated MAECs infected with vehicle or 0.1 MOI PR/8 showed that treatment of the cells with 20 µM Q-VD-Oph diminished IAV-induced caspase activity as shown by reduced cleavage of caspase 3, which served as a marker **(**Fig. 3A**)**. We have previously reported that Bik-activated caspase 3 is involved in the cleavage of viral proteins and in promoting viral replication [12]. Consequently, to test the hypothesis that Bik is involved in activating overall caspase activity, we compared *bik*-sufficient and *bik*-deficient MAECs and evaluated viral replication in the presence and absence of pan-caspase inhibitor. Compared with the vehicle-treated group, treatment with Q-VD-Oph caused ~ 1 log reduction in viral titer in differentiated MAECs from *bik*^*+/+*^ mice **(**Fig. 3B**)**. Likewise, *bik*^*-/-*^ MAECs treated with Q-VD-Oph showed ~ 1 log reduction in viral titer as compared with control **(**Fig. 3C). However, in the absence of Q-VD-Oph treatment, the viral titers in *bik*^*-/-*^ MAECs were lower by ~ 3 logs than in *bik*^*+/+*^ MAECs. Hence, our findings suggests that the pan-caspase inhibitor diminished IAV replication in MAECs in a Bik-independent manner, implying that other proteases not activated by Bik may be responsible for the further reduction in viral replication. Further, the robust reduction in virus replication in *bik*-deficient cells in the absence of Q-VD-Oph treatment signifies that additional mechanism(s) that are independent of caspases might be involved in Bik-mediated replication of IAVs. We also found that both Q-VD-Oph-treated and *bik*-deficient cells showed higher TEER values, a measure of integrity of epithelial cell integrity that encompasses tight junctions and epithelial cell viability, compared with the respective controls when infected with IAV. Q-VD-Oph treated cells showed lower decline in TEER values when infected with IAV at 48- and 72-hpi (Supplementary Fig. 1). Similarly, we have shown in our previous studies that *bik*-deficient cells infected with IAV had lower decline in TEER values compared with WT cells [12]. We found that while the there was no significant differences in the TEER values between WT and *bik*^*-/-*^ cells at 0 hpi, the *bik*^*-/-*^ cells showed significantly lower decline in TEER values compared with the WT cells both at 48- and 72-hpi (Supplementary Fig. 2).

### Treatment with the pan-caspase inhibitor mitigated lung inflammation and IAV-induced lethality

**Fig. 4:**
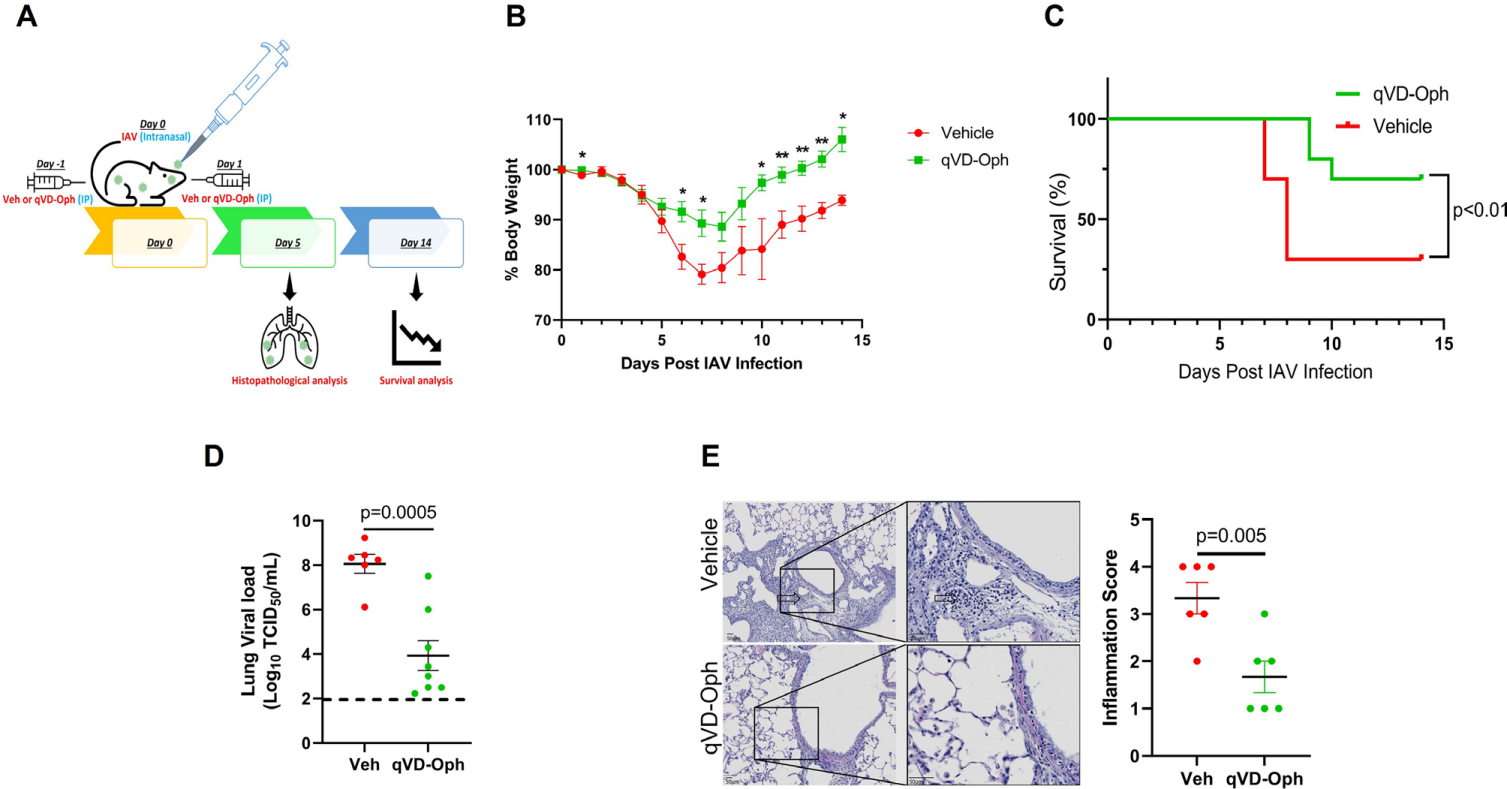
Pan-caspase inhibitor reduced lung inflammation and IAV-induced mortality. **(A) **Six to eight weeks old mice were treated with vehicle control or 20 mg/kg body weight Q-VD-Oph intraperitoneally on day − 1 and day 1 of intranasal infection with 100 pfu IAV (H1N1) PR/8/34 strain in 50 µl PBS and were monitored for **(B)** the percent change in body weight and **(C)** survival over a period of 14 days post-infection. n = 10/group; N = 2. **(D)** Lung viral load was analyzed using the median tissue culture infectious dose (TCID_50_) 5 days post-infection. The lowest detection limit of the TCID_50_ assay was approximately 10^2 and this has been shown as a **dotted line**. n = 6–8/group. **(E)** Microscopic evaluation of vehicle control or Q-VD-Oph-treated lung sections stained with hematoxylin and eosin for histopathological analysis at 5 days post-infection and quantification of inflammation score. The open arrows indicate inflammatory cell infiltration in the alveoli and septa. n = 6/group, Scale bar = 50 μm, error bars indicate mean ± SEM; p < 0.05 was considered significant

Treatment with Q-VD-Oph suppressed broad-spectrum caspase activities, including caspase 1, 2, 3, 6, 8, 9, and 12 in various disease mouse models [49–52]. Therefore, we injected mice intraperitoneally with vehicle or 20 mg/kg body weight Q-VD-Oph and infected them with a lethal dose (100 pfu) of PR/8 intranasally **(**Fig. 4A**)**. We found that while mice treated with vehicle showed drastic reduction in body weight, Q-VD-Oph-treated mice lost less weight and most of them recovered their lost body weight by 14 dpi **(**Fig. 4B**)**. Survival analysis showed that significantly more vehicle-treated mice succumbed to IAV infection as compared with Q-VD-Oph-treated mice **(**Fig. 4C**)**, suggesting that loss of overall caspase activity leads to reduced susceptibility to IAV infection. Additionally, to further evaluate viral replication, we collected the lungs of infected mice and measured virus titers and inflammation at 5 dpi and observed a significant reduction in viral load **(**Fig. 4D**)** and inflammation **(**Fig. 4E**)** in the lungs of Q-VD-Oph- compared with vehicle-treated mice. Semiquantitative histopathological evaluation of lung inflammation and tissue damage was performed by a trained pathologist in a blinded fashion. The lungs from qVD-Oph-treated mice showed reduced alveolar and/or bronchial degeneration, necrosis, and infiltration of inflammatory cells in the alveolar septa. Inflammation score was evaluated based on the percentage of the lung affected, alveolar and/or bronchial degeneration and necrosis, and the infiltration of inflammatory cells **(**Fig. 4E**)**.

### Treatment with the pan-caspase inhibitor reduced cytoplasmic export of the vRNPs and cleavage of viral HA and NP

**Fig. 5:**
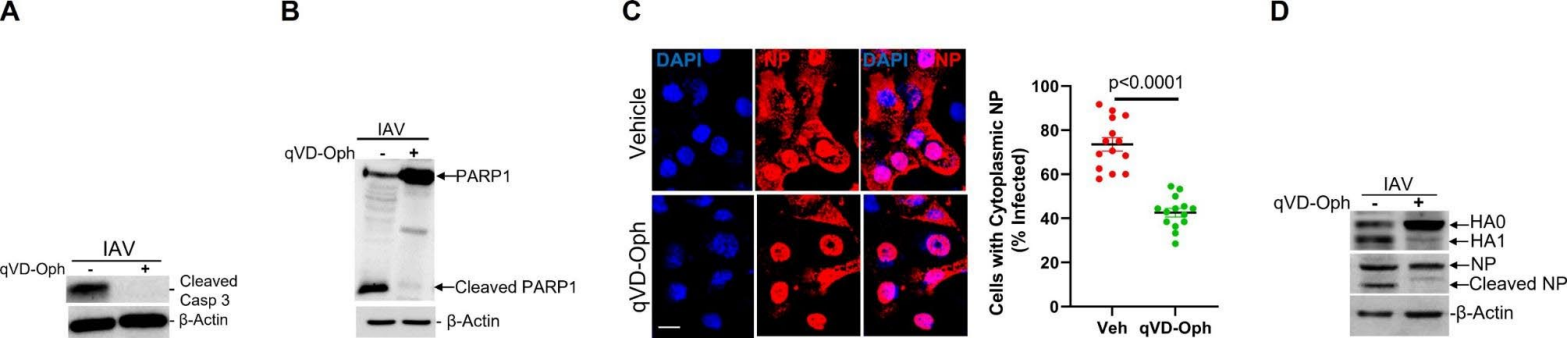
Pan-caspase inhibitor inhibits nucleo-cytoplasmic translocation of IAV-nucleoprotein (NP). **(A)** Human airway epithelial cells were infected with 0.1 MOI IAV (H1N1) PR/8/34 strain and treated with vehicle control or 20 µM Q-VD-Oph. The inhibitors were added on apical and basal sides 3 h prior to viral infection. The inhibitors were removed from the apical side but were kept with media in the basal side for the whole experimental period. Protein lysates were analyzed for **(A)** caspase 3 and **(B)** PARP1 cleavages by Western blot. **(C)** Cells were subjected to immunostaining using a specific anti-viral NP antibody and secondary antibody conjugated to Dylight-649 24 h after infection. Cells were mounted with DAPI containing Fluormount-G for nuclear staining and analyzed with fluorescent microscopy. Percentage of IAV-infected cells with cytoplasmic localization were analyzed. Experiments were performed in triplicates, and the localization of viral NP in infected cells was quantified by counting at least 100 cells per experiment. n = 14/group, Error bars indicate mean ± SEM. Scale bar = 10 μm. **(D)** Protein lysates were analyzed for cleavage of HA and NP cleavage by Western blot. Experiments were performed in triplicates and the immunoblots are representative of at least three separate experiments. Q-VD-Oph, quinoline-Val-Asp-difluorophenoxymethylketone. p < 0.05 was considered significant

IAV-induced caspase activation causes a widening of nuclear pores to facilitate passive transport of vRNP to ensure efficient production of infectious virus progeny [24, 25]. Viral NP acts as a shuttle for viral genomic segments from the nucleus to the budding sites at the plasma membrane, and localization during the virus replication cycle affects virus titers. Treatment with Q-VD-Oph inhibited IAV-induced cleavage of caspase 3 **(**Fig. 5A**)** and PARP1 **(**Fig. 5B**)**, confirming q-VD-OPh-driven loss of caspase activities in HAECs. Interestingly, while efficient NP migration to the cytoplasm was readily detected at 24 hpi in vehicle-treated cells, treatment with Q-VD-Oph significantly inhibited the cytoplasmic accumulation of the vRNPs. We observed significantly decreased cytoplasmic accumulation of viral NP **(**Fig. 5C**)**, with approximately 75% of infected cells showing cytoplasmic localization of viral NP in vehicle-treated cells compared with about 43% in Q-VD-Oph-treated cells **(**Fig. 5C**)**. These findings support a link between caspase activity and nucleo-cytoplasmic shuttling of vRNPs. Furthermore, loss of caspase activity blocked cleavage of viral proteins, HA and NP **(**Fig. 5D**)**, which might affect the proper assembly of progeny virions and efficient viral replication and pathogenicity.

## Discussion

The present study compared caspases 2, 3, 6, and PARP1 using specific inhibitors to make direct comparison of their roles in promoting viral replication. Although inhibition of each protease tested caused significant reduction in viral replication in the AECs, we found that, among the caspase inhibitors tested, caspase 6 led to the most efficient reduction of viral replication in the AECs. Interestingly, compared with the caspase inhibitors screened, PARP1 inhibition was found to be the most efficient in diminishing viral replication. In the absence of treatment with caspase inhibitors, *bik*-deficiency caused about 3 logs reduction in the viral titer, while overall caspase inhibition using Q-VD-Oph under *bik*-deficient conditions further decreased viral replication. In vivo, treatment with Q-VD-Oph provided protection from IAV-induced lung inflammation and lethality. Mechanistically, Q-VD-Oph prevented nucleo-cytoplasmic shuttling of viral NP and cleavage of HA and NP in human AECs.

According to the predicted caspase cleavage motifs, a single caspase can target multiple viral proteins [13]. Moreover, different caspases may target identical cleavage motifs on the same viral proteins [13]. These imply that inhibiting multiple caspases and PARP1 may be required to attain effective inhibition of viral replication. In this study, we found that inhibiting caspase 2 or 6 diminished viral replication in the AECs significantly, suggesting their involvement in IAV replication and corroborating previous reports on caspase 3 inhibition [11, 21, 22]. Caspase 2 has cleavage motifs on HA, M2, NS1, and PA proteins, whereas caspase 6 has cleavage motifs on M2, NA, NP, PA, and PB1. Although these caspases share identical cleavage motifs on PA and M2 proteins, caspase 6 targets more viral proteins than caspase 2 [13], Thus, it is possible that the additional target viral proteins for caspase 6 contribute for the more efficient inhibition of viral replication by caspase 6 inhibitor. Whether the caspase 6 inhibitor is specific to caspase 6 or it also inhibits other caspases including caspase 2 is not clear. Future studies are needed to define the role of caspase 2-mediated cleavage of viral proteins in IAV replication and pathogenesis. Interestingly, a recent study showed that mice deficient in caspase 6 were more susceptible to IAV infection. They found that IAV replication was not affected in Casp6^–/–^ bone marrow-derived macrophages (BMDMs), suggesting that caspase 6 regulates NLRP3 inflammasome activation independently of NLRP3 priming and viral replication in BMDMs [53]. Whether caspase 6 affects viral replication in a cell type-dependent manner or facilitates viral replication through cleavage of viral proteins only or by other immunopathogenic mechanisms in vivo needs further investigation. Considering its broad-spectrum nature, one would expect more decline in viral titer when cells are treated with Q-VD-Oph compared with the individual inhibitors. However, our current data shows otherwise, which could possibly be because of some alternate mechanism that is triggered when the overall caspase activity is blocked, which warrants further investigation.

Previous studies showed that PARP1 is required for IAV replication. Depletion of PARP1 in HEK293T cells using RNA-interference led to a significant reduction in infectious IAV replication [31]. PARP1 is required for viral RNA-dependent RNA polymerase activity of human H1N1 and avian-derived H5N1 viruses [30, 31] and controls viral HA-induced degradation of host IFNAR [16], which in turn affects viral replication. Several PARP1 inhibitors are approved as anticancer drugs for pathogenic variants of *BRCA1* and *BRCA2* [54]. *BRCA1/2*-deficient cancer cells depend on PARP1 for backup DNA repair leading to selective killing of these cancer cells by targeting PARP1. Recently approved PARP1 inhibitors for *BRCA1/2*-linked ovarian cancer (OC) produced major response rates for patients with *BRCA1/2*-linked advanced OCs [55, 56]. Since PARP1 is a downstream substrate of caspases, it is tempting to speculate that its inhibition alone could be identical to caspase inhibition, but it is also possible that PARP1 inhibitor together with caspase inhibitors may provide synergistic effect to efficiently dampen viral replication.

We previously reported that Bik-activated caspase 3 is involved in cleavage of viral protein thereby promoting viral replication [12]. However, the present study demonstrated that treatments of differentiated cultures with Q-VD-Oph led to 1 log reduction in viral titers both under *bik*^*+/+*^ and *bik*^*−/−*^ conditions. Interestingly, in the absence of treatment with Q-VD-Oph, compared with *bik*^*+/+*^ MAECs, *bik*-deficiency alone caused about 3 logs reduction in viral titer. This implies that other proteases not activated by Bik might be responsible for further reduction in viral replication in *bik*^*−/−*^ cells treated with Q-VD-Oph. Additionally, the robust reduction in viral titer in *bik*^*−/−*^ MAECs in the absence of Q-VD-Oph treatment suggested that Bik might promote viral replication through mechanism(s) that are independent of caspases and PARP1 activation. At least 14 caspases have been identified in mammalian cells with their roles ranging from apoptosis and inflammation [57] to promoting viral replication [13]. However, only a few of the caspases have been studied for their role in IAV replication and pathogenesis. Whether Q-VD-Oph sufficiently inhibits all the caspases that might be involved in IAV replication remains to be elucidated.

We previously showed that Bik activates not only caspase 3, but also lysosomal cysteine proteases, cathepsin B and D, to promote apoptotic cell death in the AECs [48]. Cathepsins [58, 59], and the transmembrane protease TMPRSS2 [60–64] have been implicated in the replication of IAV by cleaving and activating viral proteins. In human and murine respiratory cells, TMPRSS2 is a major activating protease of IAV HA subtypes [60] resulting in the spread of human IAVs of subtype H1 and H2, zoonotic H7N9 virus, and avian H10 virus in mice [61–64]. Whether Bik is involved in activating TMPRSS2 and whether Bik*-*activated cathepsins [12] plays a role in IAV replication and pathogenicity still remains to be determined.

In vivo, reduced viral load and the resulting inflammation in the lungs of mice treated with Q-VD-Oph facilitated survival of these mice even after infection with a lethal dose of IAV. These findings portray that reducing the overall caspase activity is sufficient to diminish the severity of IAV-induced lung inflammation and protects mice from virus-induced mortality. Multiple caspase cleavage motifs in virus proteins could be involved in virus reproduction and in host pathways of infection immunopathogenesis. While our results suggest that caspases are involved in host defense against IAV infection, whether blocking a single caspase or a combination of caspase and/or PARP1 is sufficient to reduce viral replication and pathogenicity in vivo remains to be seen.

**Fig. 6:**
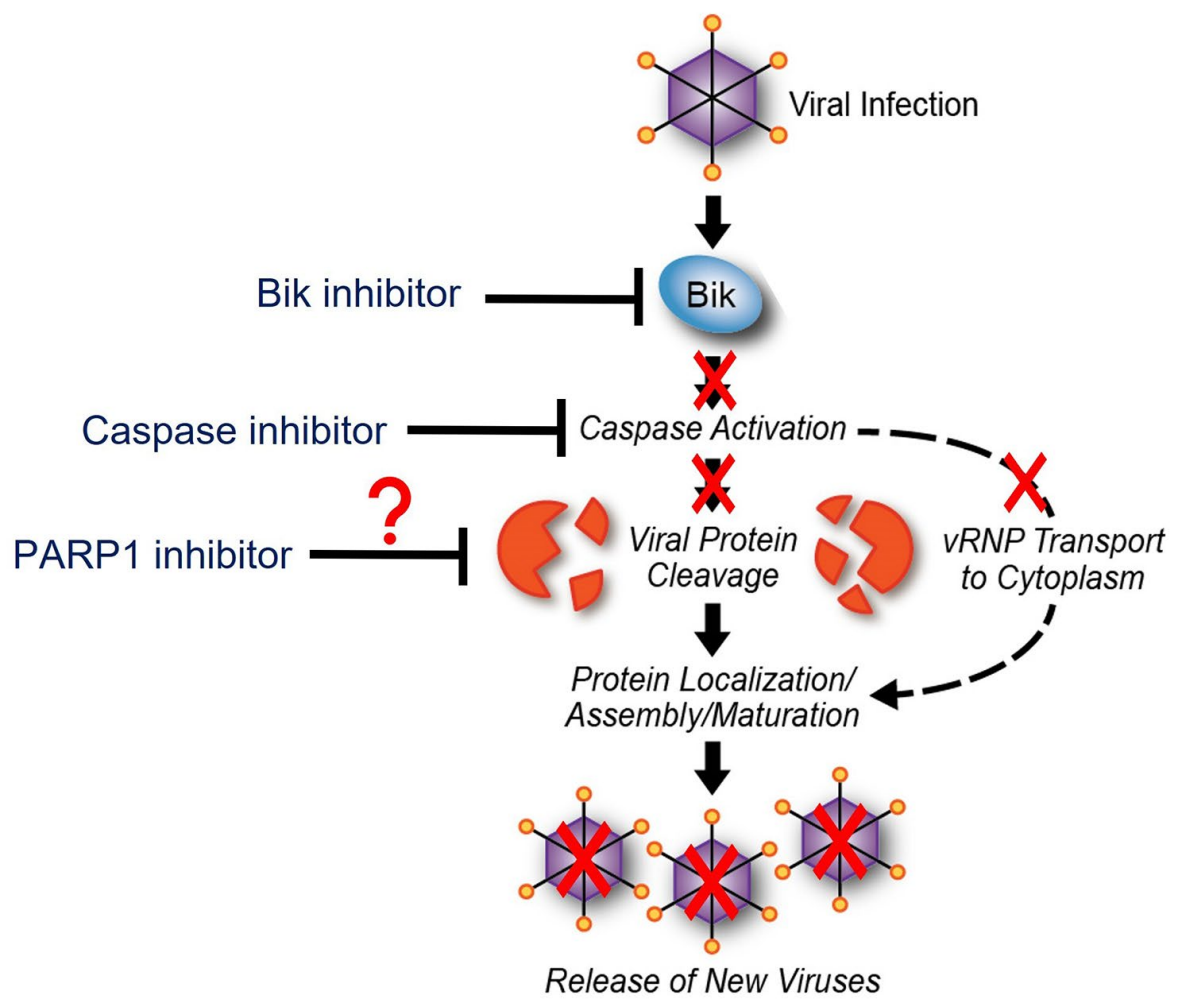
Proposed mode of action. We hypothesize that blocking Bik or inhibiting caspase(s) prevents nucleo-cytoplasmic transport of viral ribonucleoprotein (vRNP) and cleavage of viral proteins. These may impair proper assembly of progeny virions resulting in reduced viral replication. It remains to be elucidated whether PARP1 promotes cleavage of viral proteins and nucleo-cytoplasmic transport of vRNP as highlighted by the question mark

IAVs are structured into ribonucleoprotein segments consisting of viral RNA and viral proteins, the major one being NP, a target of caspase cleavage [12]. IAV-induced caspase activation causes a widening of nuclear pores to facilitate passive transport of vRNP to ensure efficient production of infectious virus progeny [24, 25]. In this study, we found that cytoplasmic accumulation of NP was significantly impaired in human AECs treated with Q-VD-Oph. We previously reported that *bik*-deficiency inhibited caspase 3 cleavage to impair cytoplasmic export of vRNP, suggesting the potential for impaired shuttling of vRNP to the cytoplasm due to *bik-*deficiency [12]. Similarly, another pro-apoptotic protein Bax regulates nucleo-cytoplasmic shuttling of vRNP by activating caspases. Bax^-/-^ caused a retention of IAV NP within the nucleus, suggesting that Bax-mediated caspase activation is involved in nucleo-cytoplasmic shuttling of vRNP [7]. Consistent with caspase activation being important, increased expression of Bcl-2 blocks caspase activation and leads to nuclear retention of vRNP complexes that impairs assembly of progeny virions and marked reduction in titers of infectious virus [11, 12, 65]. Possibly, IAVs use a strategy where caspases regulate vRNP export by increasing the diffusion limit of nuclear pores to allow passive diffusion of vRNP [24] **(**Fig. 6**)**.

## Conclusions

Our findings depict variable response in viral replication in the AECs between different proteases and PARP1; and reveals that the loss of overall caspase activity *via* treatment with pan-caspase inhibitor diminished IAV-induced lung inflammation and mortality in vivo. Further, while caspase inhibition reduced viral titers by ~ 1 log in *bik*^*-/-*^ MAECs, the robust reduction in viral titer in *bik*^*-/-*^ MAECs in the absence of Q-VD-Oph treatment suggested that Bik promotes viral replication through mechanism(s) that are independent of caspases. More detailed understanding of the role of proteases, PARP1, and possibly cathepsins involved in IAV replication may provide potential novel drug targets.

## Electronic supplementary material

Below is the link to the electronic supplementary material.

Supplementary Material 1

## Data Availability

The datasets used and/or analyzed during the current study are available from the corresponding author upon reasonable request.

## Abbreviations

AECs: Airway epithelial cells
ALI: Air-liquid interface
BEGM: Bronchial epithelial growth medium
*bik*^*−/−*^: Bik knockout
*BMDMs*: Bone marrow-derived macrophages
Caspases: Cysteine Aspartate-Specific Proteases
DMEM: Dulbecco’s Modified Eagle Medium
dpi: Days post-infection
ECL: Enhanced chemiluminescence
FBS: Fetal bovine serum
HA: Hemagglutinin
HAEC: Human airway epithelial cells
HCV: Hepatitis C Virus
HIV: Human immunodeficiency virus
hpi: Hours post-infection
IAV: Influenza A virus
IFNAR: Type I interferon receptor
IP: Intraperitoneal
M2: Matrix protein 2
MAECs: Mouse airway epithelial cells
MDCK: Madin Darby canine kidney
MEM: Minimum essential medium
NA: Neuraminidase
NP: Nucleoprotein
NS: Nonstructural
OC: Ovarian cancer
PARP1: Poly (ADP-ribose) polymerase
PBS: Phosphate buffered saline
pfu: Plaque-forming unit
PR/8/34: A/Puerto Rico/8/1934 (H1N1) strain
Q-VD-Oph: Quinoline-Val-Asp-difluorophenoxymethylketone
RT: Room temperature
SAGM: Small airway epithelial cells growth media
SEM: Standard error mean
TCID_50_: Median tissue culture infectious dose
TEER: Trans-epithelial electrical resistance
vRNP: Viral ribonucleoprotein
WT: Wild-type
