# Correction: Independent role of caspases and Bik in augmenting influenza A virus replication in airway epithelial cells and mice

**DOI:** 10.1186/s12985-023-02060-9

**Published:** 2023-05-15

**Authors:** Sourabh Soni, Stephanie Walton-Filipczak, Richard S. Nho, Yohannes Tesfaigzi, Yohannes A. Mebratu

**Affiliations:** 1 Division of Pulmonary, Critical Care, and Sleep Medicine, Department of Internal Medicine, The Ohio State University Wexner Medical Center, Columbus, OH USA; 2 Lovelace Respiratory Research Institute, Albuquerque, NM USA; 3 New Mexico Department of Game and Fish, Santa Fe, NM USA; 4 Division of Pulmonary and Critical Care Medicine, Department of Medicine, Brigham and Women’s Hospital, Harvard Medical School, Boston, MA USA

**Correction: Virology Journal (2023) 20:78**

10.1186/s12985-023-02027-w

Following publication of the original article [1], the authors would like to update the Fig. 4D.

The updated Fig. 4 is given below.

**Fig. 4:**
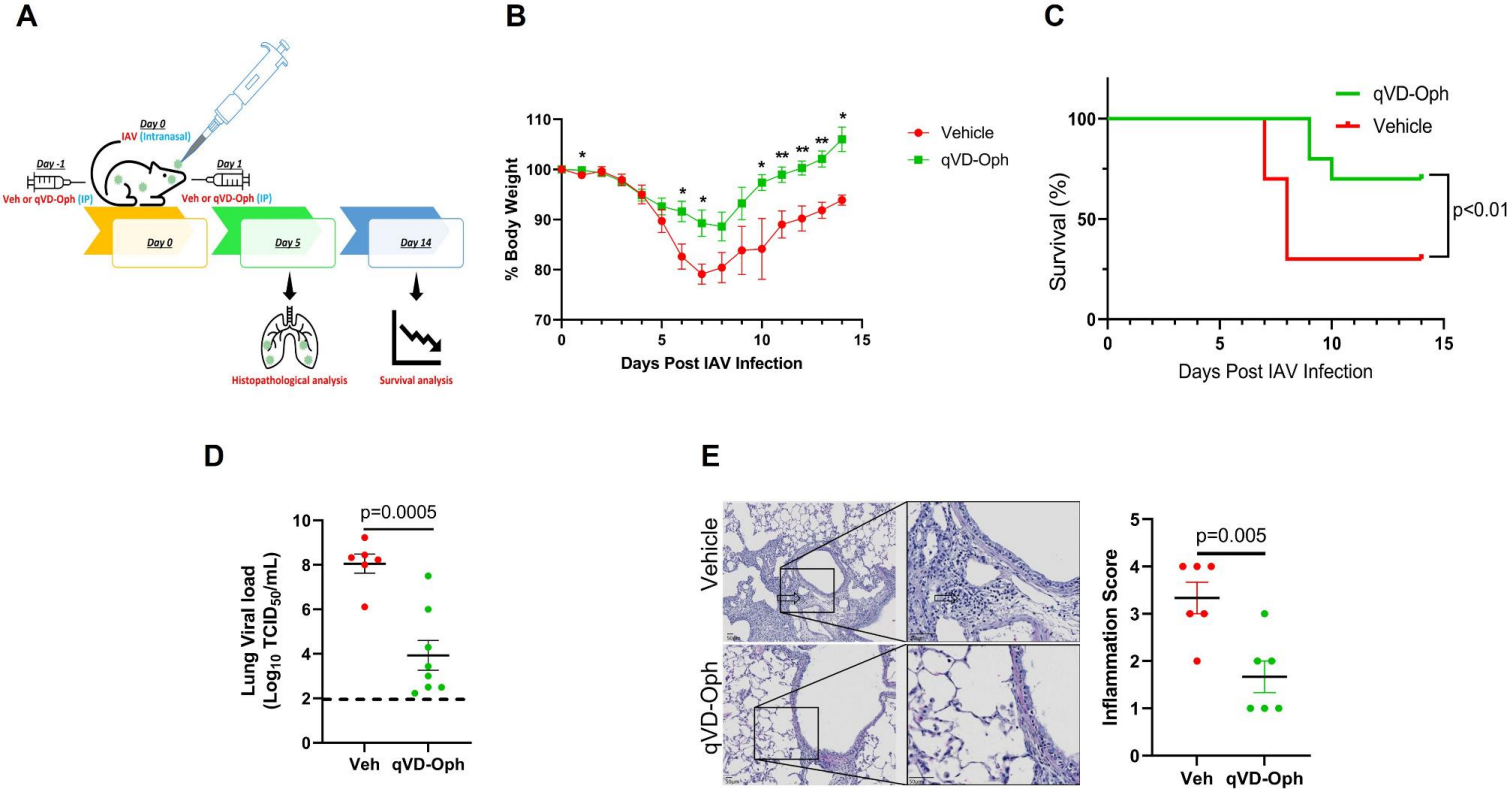
Pan-caspase inhibitor reduced lung inflammation and IAV-induced mortality. **(A)** Six to eight weeks old mice were treated with vehicle control or 20 mg/kg body weight Q-VD-Oph intraperitoneally on day − 1 and day 1 of intranasal infection with 100 pfu IAV (H1N1) PR/8/34 strain in 50 µl PBS and were monitored for **(B)** the percent change in body weight and **(C)** survival over a period of 14 days post-infection. n = 10/group; N = 2. **(D)** Lung viral load was analyzed using the median tissue culture infectious dose (TCID_50_) 5 days post-infection. The lowest detection limit of the TCID_50_ assay was approximately 10^2 and this has been shown as a dotted line. n = 6–8/group. **(E)** Microscopic evaluation of vehicle control or Q-VD-Oph-treated lung sections stained with hematoxylin and eosin for histopathological analysis at 5 days post-infection and quantification of inflammation score. The open arrows indicate inflammatory cell infiltration in the alveoli and septa. n = 6/group, Scale bar = 50 μm, error bars indicate mean ± SEM; p < 0.05 was considered significant The original article has been corrected
