# Supplementary material for: Independent role of caspases and Bik in augmenting influenza A virus replication in airway epithelial cells and mice
Source: Virol J. 2023 Apr 24;20:78. doi: 10.1186/s12985-023-02027-w (PMC10127399; doi:10.1186/s12985-023-02027-w)
Supplement: Supplementary file 1 — Supplementary Material 1 [file 12985_2023_2027_MOESM1_ESM.docx]

**Independent Role of Caspases and Bik in Augmenting Influenza A Virus Replication in Airway Epithelial Cells and Mice**

Sourabh Soni, Stephanie Walton-Filipczak, Richard S. Nho, Yohannes Tesfaigzi, and Yohannes A. Mebratu

**Supplementary Data**


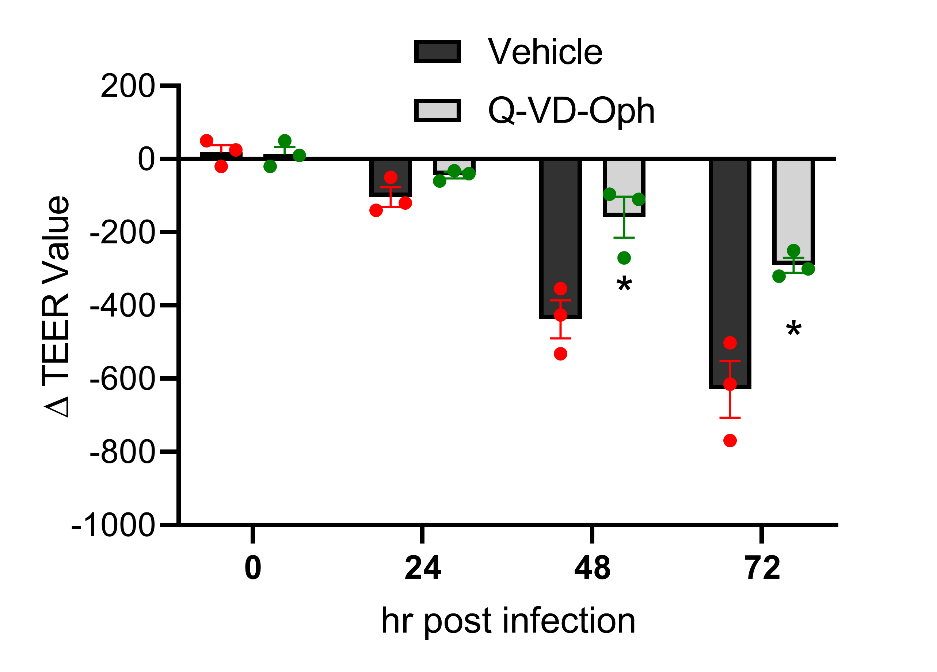


**Figure S1:** Graphical representation of transepithelial electrical resistance (TEER) values in vehicle or Q-VD-Oph treated differentiated airway epithelial cells (AECs) infected with IAV PR/8 at different time points post-infection. n = 3 per group. *P < 0.05


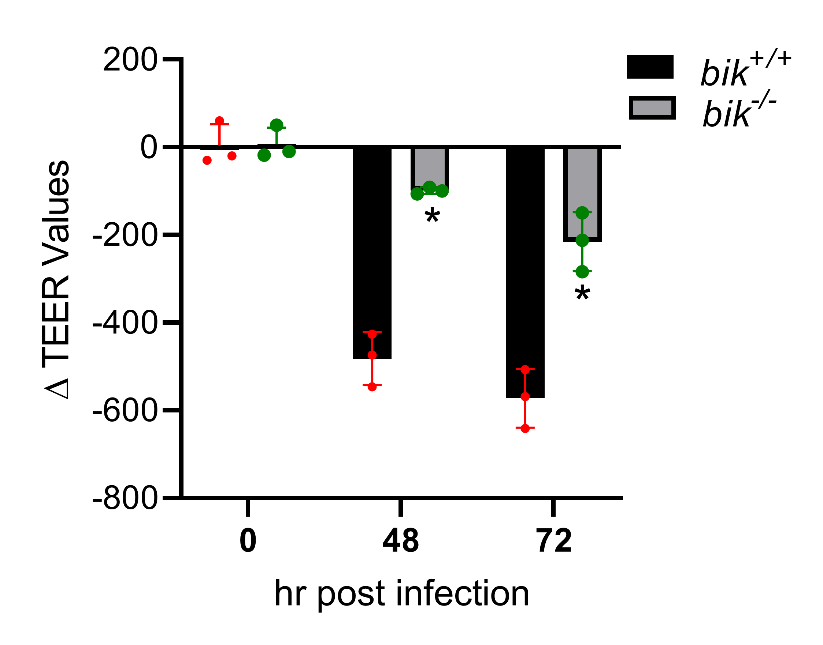


**Figure S2:** Graphical representation of transepithelial electrical resistance (TEER) values in wild-type (*bik^+/+^*) or *bik*-deficient (*bik^-/-^*) differentiated airway epithelial cells (AECs) infected with IAV PR/8 at different time points post-infection. n = 3 per group. *P < 0.05

**Figure S3:** Relative protein concentration of Cleaved Caspase 6/Full length Caspase 6 is indicated for the caspase 6 blot. Data are shown as mean ± SEM; n = 2 per group.
